# Supplementary material for: Power-law population heterogeneity governs epidemic waves
Source: PLoS One. 2020 Oct 14;15(10):e0239678. doi: 10.1371/journal.pone.0239678 (PMC7556502; doi:10.1371/journal.pone.0239678)
Supplement: Attachment — Submitted filename: nbbhj.pdf [file pone.0239678.s001.pdf]

Referee's report on the manuscript "Powe-law population heterogeneity governs epidemic waves" by Jonas Neipel, Jonathan Bauermann, Stefano Bo, Tyler Harmon, and Frank Jülicher

In the paper under review, the authors give a detailed study of the classical SIR model for epidemics in the case when the heterogeneity of the susceptible population is allowed. The main focus is made on the case where the initial susceptibility distribution has gamma density with parameter α. This, in particular, leads to the time-dependent reproduction number $R(t)$, decreasing as hyperbolic function, i.e. $R(t) = (\bar{x}(t))^{1+\alpha}R_0$. The authors provide quantitative properties of the model, such as the herd immunity level, the final size of epidemics, etc. An important conclusion is that, in the heterogeneous population, the herd immunity level can be much lower than in homogeneous case (typically 60%).

In my opinion, the subject and results of the paper are very interesting, the paper is well written and I recommend it for publishing in the journal. Below, please find a list of comments.

Comments:

1. In the models for SARS-CoV-2 epidemics, the SEIR model is in widespread use, where E stands for the "exposed" compartment. I wonder, if it is possible to relate it with the generalized SIR model in (1)–(2) with time-varying parameters?

2. The model in eqs. (1)–(2) is not very clearly explained. The classical SIR model assumes three components – susceptible (S), infected (I) and recovered (R). Whereas eqs. (1)–(2) of the paper does not include part R. Also, it is slightly unclear the meaning of $\bar{x}$. It would be useful to explain what does it mean "dimensionless average susceptibility". Usually the coefficient at IS/N is called "infection rate" and can admit values larger (or smaller) than 1.

3. p. 5. It is mentioned that time-varying β could correspond to seasonal changes or mitigation measure. For illustrative purposes, it would be nice to see concrete forms of $\beta(t)$ in such cases.

4. It is well-known that SIR-type compartment models are rather sensitive to initial conditions. It would would be good to see some discussion on this sensitivity. At least, how the graphs will change if you take other values than $I_0 = 10$.

5. p. 7, l. -9. Since s depends on both x and t, I would recommend to write $S(t) = \int_0^\infty dxs(x,t)$. Similarly, in eq. (10), as the right-hand side depends on t, better to write $\bar{x}(t) = \frac{1}{S(t)} \int_0^\infty dx\, xs(x,t)$.

6. p. 7, l. -2. The meaning of the variable τ ("a measure for how far the epidemic has advanced") should be explain better.

7. p, 8, eq. (12). Strictly speaking, eq. (12) gives a *density function*, not distibution. Also, you may wish to add that $\alpha > 0$ and to write a precise form

$$s_0(x) = \frac{\alpha^\alpha}{\Gamma(\alpha)} x^{-1+\alpha} e^{-\alpha x}, \; x > 0.$$

(The symbol '$\sim$' usually means asymptotic equivalence.) Also, I wonder if it would be possible to introduce additional flexibility in the initial susceptibility by introducing two-parameter gamma distribution with density

$$s_0(x) = \frac{\lambda^\alpha}{\Gamma(\alpha)} x^{-1+\alpha} e^{-\lambda x} \, ?$$

Minor comments:

1. p. 3, l. -11. Delete 'the'.
2. p. 8, eq. (13). Please add more details how this equality is obtained.
3. p. 9. Please add more details how eqs. (18) and (19) are obtained.
4. p. 22. It seems that the Lambert W-function should be defined by $W(z)e^{W(z)} = z$.
